# Cyclodextrins as multifunctional excipients: Influence of inclusion into β-cyclodextrin on physicochemical and biological properties of tebipenem pivoxil

**DOI:** 10.1371/journal.pone.0210694

**Published:** 2019-01-25

**Authors:** Magdalena Paczkowska, Daria Szymanowska-Powałowska, Mikołaj Mizera, Dominika Siąkowska, Wioletta Błaszczak, Hanna Piotrowska-Kempisty, Judyta Cielecka-Piontek

**Affiliations:** 1 Department of Pharmacognosy, Poznan University of Medical Sciences, Święcickiego, Poznan, Poland; 2 Department of Biotechnology and Food Microbiology, Poznan University of Life Sciences, Wojska Polskiego, Poznan, Poland; 3 Institute of Animal Reproduction and Food Research of Polish Academy of Sciences, Tuwima, Olsztyn, Poland; 4 Department of Toxicology, Faculty of Pharmacy, Poznan University of Medical Sciences, Dojazd, Poznan, Poland; Northeastern University, UNITED STATES

## Abstract

A novel approach for drug design based on the oral carbapenem analog tebipenem pivoxil (TP) has been proposed. The formation of the tebipenem pivoxil-β-cyclodextrin (TP-β-CD) complex resulted in changes concerning physicochemical properties of TP, which is significant for planning the development of an innovative pharmaceutical formulation as well as in the modifications of biological activity profile of the studied delivery system. The inclusion of TP into β-cyclodextrin (β-CD) was confirmed by spectral (infrared and Raman spectroscopies) and thermal method (differential scanning calorimetry). Precise indications of TP domains responsible for interaction with β-CD were possible through a theoretical approach. The most important physicochemical modifications obtained as an effect of TP inclusion were changes in solubility and its rate depending on acceptor fluids, and an increase in chemical stability in the solid state. Biologically essential effects of TP and β-CD interactions were decreased TP permeability through Caco–2 cell monolayers with the use of efflux effect inhibition and increased antibacterial activity. The proposed approach is an opportunity for development of the treatment in resistant bacterial infections, in which along with physicochemical modifications induced by a drug carrier impact, a carrier synergy with a pharmacological potential of an active pharmaceutical substance could be used.

## Introduction

The carbapenems, which belong to the β-lactam antibiotics, are unique because of the broad spectrum of bacteriostatic activity due to the relative resistance to hydrolysis by most β-lactamases [1]. Therefore, carbapenems are antibiotics which nowadays, in the era of widespread antibiotic resistance, can be used as one of the very few options to treat severe hospital-acquired infections [2]. The greatest limitations of their application are: (1) carbapenem resistance, mainly among Gram-negative pathogens, which involve active expulsion out of the periplasmic space after their entrance into bacteria, (2) low bioavailability connected with hydrophilic properties, and (3) significant degradation in the gastric environment in the solid state [3–5].

Due to the above-mentioned limitations, especially susceptibility to gastric environment, all carbapenem analogs, excluding tebipenem pivoxil (TP), must be administered parenterally [6]. TP is the first carbapenem analog recommended for oral administration as a prodrug [7]. TP shows activity against the majority of Gram-positive and Gram-negative bacteria such as methicillin-resistant *Staphylococcus aureus* (MRSA), methicillin-resistant *Staphylococcus epidermidis*, *Enterococcus faecalis*, *Escherichia coli*, *Klebsiella pneumoniae*, *Enterobacter aerogenes* and *Pseudomonas aeruginosa* [8].

Esterification of the acidic group at C-2 bicycle 4:5 fused rings in tebipenem increases the lipophilicity of a molecule and also constitutes a priority location for acid catalyzed hydrolysis. After the administration of tebipenem pivoxil, 54–75% of the dose is excreted in the urine as tebipenem [9–10]. Tebipenem pivoxil is considered to exhibit higher intestinal absorption than other β-lactam prodrugs (e.g. cefditoren pivoxil, cefcapene pivoxil, and cefetamet pivoxil). The mechanism of drug permeability through biological barriers includes passive diffusion, active transport, paracellular and efflux pathways. Knowledge about the absorption of β-lactam antibiotics provided information that their hydrophilic forms are absorbed through passive diffusion, while prodrugs also need peptide transporters [11]. Kato *et al*. suggested that tebipenem pivoxil is transported by OATP1A2 and OATP2B1 (organic anion transporting polypeptides) and not by MDR1 action (P-glycoprotein), as reported for other β-lactam prodrugs [12].

A significant chemical instability of carbapenems, including TP, has been confirmed by susceptibility to acid-base hydrolysis as well as thermolysis in the research concerning accelerated stability studies in the solid state [13–14]. It was confirmed that the main degradation products of TP in the solid state were: a product resulting from the condensation of the substituents of 1-(4,5-dihydro-1,3-thiazol-2-yl)-3-azetidinyl] sulphanyl, both acid and ester forms of tebipenem with an open β-lactam ring observed in dry air at an increased temperature (RH = 0%, T = 393 K), acid and ester forms of tebipenem with an open β-lactam ring observed at an increased relative air humidity and increased temperature (RH = 90%, T = 333 K). During acid-base hydrolysis, tebipenem was also formed as the principal degradation product. Although the chemical instability of tebipenem pivoxil is lower than reported for acidic forms of carbapenems, it is still a significant limitation of its use. At the same time, the uncontrolled degradation of tebipenem pivoxil in the gastrointestinal tract promotes the formation of resistant strains related to the presence of its active form—tebipenem. Moreover, in light of the proven catalytic effect of selected compounds (HCO_3_^-^) on the degradation of selected carbapenems (e.g. meropenem), it might appear that there are only a limited number of excipients which fulfill the criteria of valuable stabilizers of carbapenem analogs [15].

Carbapenem analogs stabilization with the simultaneous preservation of their antibacterial activity is of great importance considering current constraints in terms of severe bacterial infections and can constitute a precious solution for future therapies.

Considering that it is necessary to stabilize carbapenem analogs and improve their permeability and antibacterial activity, cyclodextrins (CDs) may be recommended as auxiliary substances. Cyclodextrins are biopolymers that contain six, seven or eight glucose monomers, linked by α-1,4-glucose bonds, referred to as α-, β- or γ-cyclodextrins, respectively. It has been reported in a number of studies that CDs are able to form inclusion (host-guest) complexes with several antibiotics [16–19]. When used as complexing agents, CDs can also increase antibiotic’s solubility and enhance drug permeability through the membrane barrier, thus improving the bioavailability of the guest molecule, and modifying the antibacterial activity and chemical stability. The effect of β-CD on aqueous solubility and dissolution rate was evaluated in the case of cefpodoxime proxetil [17]. It was confirmed that the presence of β-CD effectively enhanced the aqueous solubility of cefpodoxime proxetil. With regards to the influence of CDs on permeability, it was noted that hydroxypropyl-β-cyclodextrin and β-cyclodextrin inclusion complexes significantly (p<0.01) increased the apparent intestinal permeability of trimethoprim by 39.8% and 56.1% respectively, when apparent permeability coefficients were determined using a Caco-2 permeability assay [19]. Antibacterial activity of cyclodextrin-included antibiotics was increased (especially for hydrophobic antibiotics), particularly against Gram-negative clinical strains [16]. It was also reported that the formation of a meropenem-β-cyclodextrin inclusion complex increased meropenem chemical stability (during stability studies in the solid state at increased relative air humidity) of meropenem, which is important for the preparation and administration of its parenteral solutions [18].

Therefore, the aim of the present work was to prepare and characterize a tebipenem pivoxil inclusion complex with β-cyclodextrin in order to achieve changes in the solubility, dissolution, chemical stability, Caco-2 permeability and antibacterial activity.

## Materials and methods

### Materials

Tebipenem pivoxil (purity >98%) was supplied by Pharmachem International Co., (China). β-cyclodextrin (purity >98%) was obtained from Sigma-Aldrich (Poland). UHPLC grade acetonitrile was supplied by Merck KGaA (Germany) and formic acid (100%) by Avantor Performance Materials (Poland). Hydrochloric acid, potassium dihydrogen phosphate, sodium chloride and potassium bromide were obtained from Avantor Performance Materials (Poland). High-quality pure water was prepared by using an Exil SA 67120 Millipore purification system (France).

### Preparation of the TP-β-CD inclusion complex

Solid inclusion complex of tebipenem pivoxil (TP) with β-cyclodextrin (β-CD) was obtained by dry mixing method [20]. TP with β-CD was grinding in the same molar ratio 1:1 with continuous stirring for 30 min. Then, the TP-β-CD complex was kept at 308 K in ambient humidity for 24 h and finally grinding to powder and stored at constant ambient humidity in the evacuated chambers at 293 K.

### The identification of the TP-β-CD inclusion complex

Identification of the TP-β-CD complex was confirmed with regards to results of spectroscopic (changes in FT-IR/Raman spectra of TP-β-CD complex in comparison with TP in free form) and thermal (changes in DSC thermograms) studies. Quantum-chemical calculations of theoretical spectra and docking of TP into the β-CD cavity were used as the support in the analysis and prediction of the formation of the complex.

#### FT-IR spectroscopy (FT-IR)

TP, β-CD and the TP-β-CD inclusion complex were obtained separately with IR grade potassium bromide at a ratio of 1:100, and IR pellets were prepared by applying 8 metric tonnes of pressure in a hydraulic press. The vibrational infrared spectra were measured between 500 and 4000 cm^-1^, with an FT-IR Bruker Equinox 55 spectrometer equipped with a Bruker Hyperion 1000 microscope. In order to analyze changes in the positions and intensity of bands in experimental spectra of the TP-β-CD inclusion complex, quantum-chemical calculations were performed based onB3LYP functional and 6-31G(d,p) as a basis set. All calculations were performed using the Gaussian 09 package and the GaussView application [21].

#### Raman spectroscopy

Raman scattering spectra were recorded with a Lab-RAM HR800 spectrometer (HORIBA Jobin Yvon) with laser excitation λ_exc_ = 633 nm (HeANe laser). In each case, the power of the laser beam at the sample was less than 1 mW to avoid damage to the sample.

#### Differential scanning calorimetry (DSC)

DSC analysis of TP, β-CD and the TP-β-CD inclusion complex was performed using a Perkin Elmer Diamond equipped with an intercooler system. Indium was used for calibration. Accurately weighed samples were placed in 60 μL sealed cells, and heated at a scanning rate of 10 K min^-1^ from 293 K to 453 K under a nitrogen purge gas with a flow rate of 20 mL min^-1^. Each run was repeated at least twice.

#### Theoretical studies

Structures of isolated molecules: TP and β-CD were optimized with Density Functional Theory (DFT) with Becke, three-parameter, Lee-Yang-Parr (B3LYP) functional and 6-31G(d,p) basis sets. Initial conformations were set equal to the crystallography structures acquired from Open Crystallography Database [22]. The optimized TP structure was docked to the β-CD structure with preserved *ab initio* calculated atomic charges. AutoDock Vina was applied to perform fast molecular docking, bounded by a docking grid spanning all over the molecule of β-CD allowing inclusion from both sides of the macromolecule [23]. Docked complexes with the greatest affinity were considered the most stable according to molecular docking with AutoDock Vina and optimized with MMFF94 (Merck Molecular Force Field 94) molecular dynamics [24]. Molecular dynamics simulations were carried out for 10ps in 300K isothermal conditions. The stability of the TP-β-CD complex was analyzed during the simulation by means of observations of potential system disintegration. Docked complexes were further investigated with semi-empirical PM6 (Parameterization Method 6). The investigation with PM6 was based on an evaluation of the different geometries of the TP and β-CD complexes acquired by longitudinally rotating TP molecule in range of 0° to 180° with a step of 30°. The molecular energies for the TP and β-CD complex were compared to reveal the most energetically favored conformer.

### Studies of physicochemical properties of the TP-β-CD inclusion complex

The changes of TP concentrations during solubility, dissolution and permeability studies were determined by using previously developed and validated HPLC-DAD method. The separation of TP in the presence of its active form, tebipenem, was done using the LC system (DionexThermoline Fisher Scientific, Germany) equipped with a high pressure pump (UltiMate 3000), an autosampler (UltiMate 3000) and a DAD detector (UltiMate 3000) with Chromeleon software version 7.0 from DionexThermoline Fisher Scientific (US). Separations were performed on a Kinetex-C18 column (100 mm × 2.1 mm, 5.0 μm). The detection of TP was performed using a diode array detector at a wavelength maxima (𝜆_max_) of 300 nm. The mobile phase consisted of a mixture of 0.1% formic acid and acetonitrile (80:20 *V*/*V*) with a mobile phase flow rate of 0.5 mL min^-1^. The column and autosampler tray were set at 298 K and 278 K, respectively.

The changes of TP concentrations were measured in order to determine the differences between TP in free and complexed form during phase-solubility, chemical stability, dissolution and permeability studies.

#### Phase-solubility studies of the TP-β-CD inclusion complex

Phase-solubility measurements were carried out according to the method of Higuchi and Connors [25]. Amount of TP (1 mmol L^-1^) was added to various concentrations of β-CD (0–10 mmol L^-1^) and the mixture was co-ground. Solvents were prepared at ambient temperature. Changes in TP concentration were measured using the HPLC-DAD method. The TP phase-solubility was obtained by plotting the solubility of TP as the function of the concentration of β-CD. Because stoichiometric balance between TP and β-CD was 1:1, the apparent stability constants (K_1:1_) were estimated from the straight line of the phase solubility diagrams according to the equation of Higuchi and Connors [25]:

$$K_{1:1}=\frac{slope}{S_{0}(1-slope)}$$ (1)

#### Dissolution of the TP-β-CD inclusion complex

Dissolution studies of TP from TP-β-CD inclusion complex were performed by using a standard paddle Agilent 708-DS Dissolution Apparatus with a 500-mL dissolution medium at 310 ± 0.5 K and 50 rpm for 60 minutes. Samples were weighed into gelatine capsules and then placed in the spring in order to prevent flotation of the capsule on the surface of the liquid. As dissolution media, both artificial gastric juice at pH 1.2 and phosphate buffer at pH 7.2 were used. At appropriate time intervals, dissolution samples (5.0 mL) were collected with the replacement of equal volumes of temperature-equilibrated media and filtered through a 0.45 μm membrane filter. Preparation of all solvents and procedures of determinations were conducted according to the requirements of Pharmacopeia guidelines [26]. The dissolved drug concentration was measured by using the HPLC-DAD method. The similarity of dissolution percentage of TP in different forms (until it achieves plateau) was established based on *f*_1_ and *f*_2_ parameters and was defined by the following equation:

$$f_{1}=\frac{\sum_{j=1}^{n}\left|R_{j}-T_{j}\right|}{\sum_{j=1}^{n}R_{j}}\times 100$$ (2)

$$f_{2}=50\times \log\left({\left(1+(\frac{1}{n})\sum_{j=1}^{n}{\left|R_{j}-T_{j}\right|}^{2}\right)}^{-\frac{1}{2}}\times 100\right)$$ (3)

in which *n* is the number of withdrawal points, *R*_j_ is the percentage dissolved of reference at time point *t*, and *T*_j_ is the percentage dissolved of the test at time point t. According to the Moore and Flanner model, the value of 0 for *f*_1_ and the value of 100 for *f*_2_ suggests that the test and reference profiles are identical. Values between 50 and 100 indicate that the dissolution profiles are similar, whereas smaller values imply an increase in dissimilarity between release profiles [27].

#### Stability studies of the TP-β-CD inclusion complex

The degradation of TP in free and complexed forms in the solid state was studied during the accelerating stability studies at an increased relative air humidity (RH ~ 76%, T = 303–343 K) and in dry air (RH = 0%, T = 333–363 K). For these tests, TP and TP-β-CD samples, corresponding to a TP content of 5.0 mg, were accurately weighed into 5 mL vials. So-prepared samples were inserted in desiccators filled with saturated solutions of inorganic salt of sodium chloride (RH ~76%) and in a sand bath placed in heat chambers for stability studies at an increased relative air humidity and in dry air, respectively. At specified time intervals, determined by the rate of degradation, the samples of TP and TP-β-CD were removed, cooled to room temperature, and then their contents were dissolved in water, quantitatively transferred into measuring flasks, and diluted with water to 25.0 mL. Determinations of changes in the concentrations of TP were then measured.

To verify k_obs_ determined for TP and TP-β-CD degradation, a parallelism test was used. The following equations were applied to establish the significant differences in stability plots:

$$t_{0}=\frac{a_{1}-a_{2}}{S_{a_{1}-a_{2}}}$$ (4)

$$S_{a_{1}-a_{2}}=\sqrt{\frac{\sum_{i=1}^{n_{1}}{\left(y_{i1}-{\bar{y}}_{i1}\right)}^{2}+\sum_{i=1}^{n_{2}}{\left(y_{i2}+{\bar{y}}_{i2}\right)}^{2}}{n_{1}+n_{2}-4}\times (\frac{1}{\sum_{i=1}^{n_{1}}{\left(x_{i1}-{\bar{x}}_{1}\right)}^{2}}+\frac{1}{\sum_{i=1}^{n_{2}}{\left(x_{i2}-{\bar{x}}_{2}\right)}^{2}})}$$ (5)

where $\sum_{i=1}^{n_{1}}{\left(y_{i1}-{\bar{y}}_{i1}\right)}^{2}$ and $\sum_{i=1}^{n_{2}}{\left(y_{i2}+{\bar{y}}_{i2}\right)}^{2}$ are the sum of the squares of the difference in the deviation of the regression curve determined experimentally.

#### Permeability studies of the TP-β-CD inclusion complex

The Caco-2 colon cancer cell line was purchased from the European Type Culture Collection. The Caco-2 cell line was maintained in phenol red-free DMEM supplemented with 10% fetal bovine serum (FBS), 2 mmol L^-1^ glutamine, penicillin (100 U mL^-1^), and streptomycin (0.1 mg mL^-1^). Cells were cultivated under standard conditions at 310 K in a humidified atmosphere containing 5% CO_2_ and 95% air. For transport assay, the Caco-2 cells were seeded at 4x10^5^ cells/cm^2^ onto transparent membranes (Millipore) (pore size 0.4 μm, growth area 0.6 cm^2^) in clusters of 12 wells (Falcon). The growth medium was changed every day until the time of use (21 days). The permeability of TP and TP-β-CD was measured under iso-pH conditions (pH 7.4A–7.4B) in the apical-to-basolateral (A–B) and basolateral-to-apical (B–A) directions. Before the initiation of transport studies and after the test, TEER (transepithelial electric resistance) was measured using a Millicell ERS-2 Epithelial Volt-Ohm Meter. Only cells with TEER values of >450 Ω were used for the assay. Test compounds were diluted in HBSS buffer (pH 7.4) at a concentration of 5 mg mL^-1^. The volume on the apical side was maintained at 400 μL and the volume on the basolateral side was 600 μL. Samples were taken from the receiver and donor side at 15, 30, 60, 90, 120 min, replaced with HBSS and run in triplicate.

#### Studies of antibacterial activity of TP in TP-β-CD inclusion complex

The applied method follows the standards of the National Committee for Clinical Laboratory Standards (NCCLS) [18]. The bacterial strains analyzed with the MICs analysis included ATCC reference strains and clinical isolates from the Institute of Laboratory Medicine (Poznan, Poland). Indicator microorganisms were cultured in soy-casein broth with yeast extract for microorganisms with increased nutritional requirements. Bacteria were cultured under aerobic conditions (300 K, 24 h). Firstly, test tubes with a liquid medium for bacteria were prepared (1 McFarland). Then decreasing concentrations were added to each test tube. Next, test tubes were inoculated with the same amount of cell suspension. After 16–18 hours of incubation at 300 K, the growth of strains was checked via turbidity increase observations. In test tubes containing less than the MIC of examined drugs, increased turbidity was observed (the cells have grown). The minimal concentration of drugs that inhibited culture growth was defined as the MIC.

## Results and discussion

In the first part, the work focused on preparing the TP-β-CD complex via a dry mixing technique [20]. The formation of the complex was a spontaneous and repeatable process. In order to identify the TP-β-CD complex, spectroscopic (FT-IR and Raman spectra coupled with DFT calculations as supporting methods) and thermal (DSC) methods were used. A possible mechanism of formation of TP-β-CD inclusion complex was proposed in the results of theoretical calculations.

### FT-IR and Raman analysis

**Fig 1:**
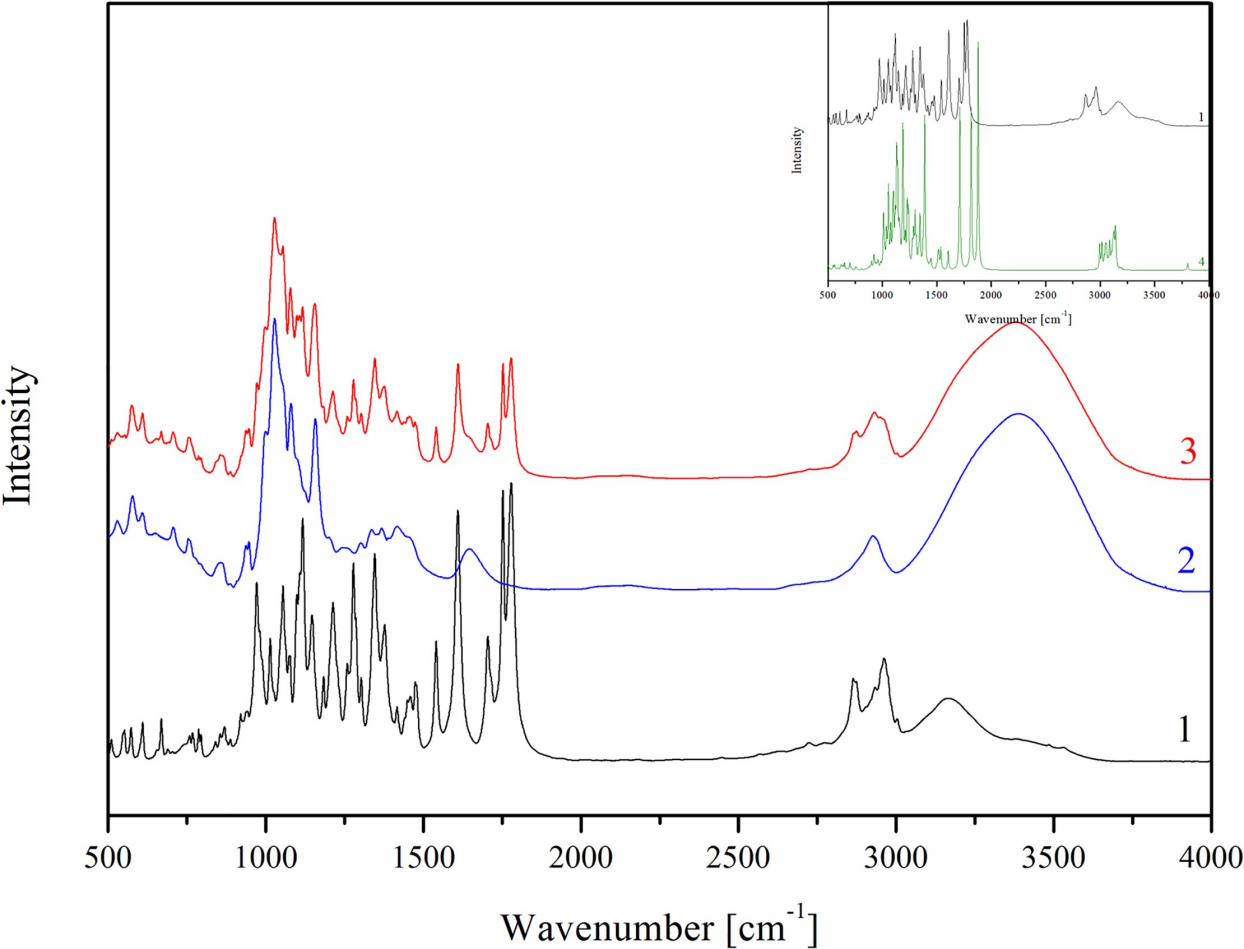
FT-IR absorption spectra for TP (1), β-CD (2), TP-β-CD inclusion complex (3) and calculated IR absorption TP spectrum (B3LYP/6-31G) (4).

**Fig 2:**
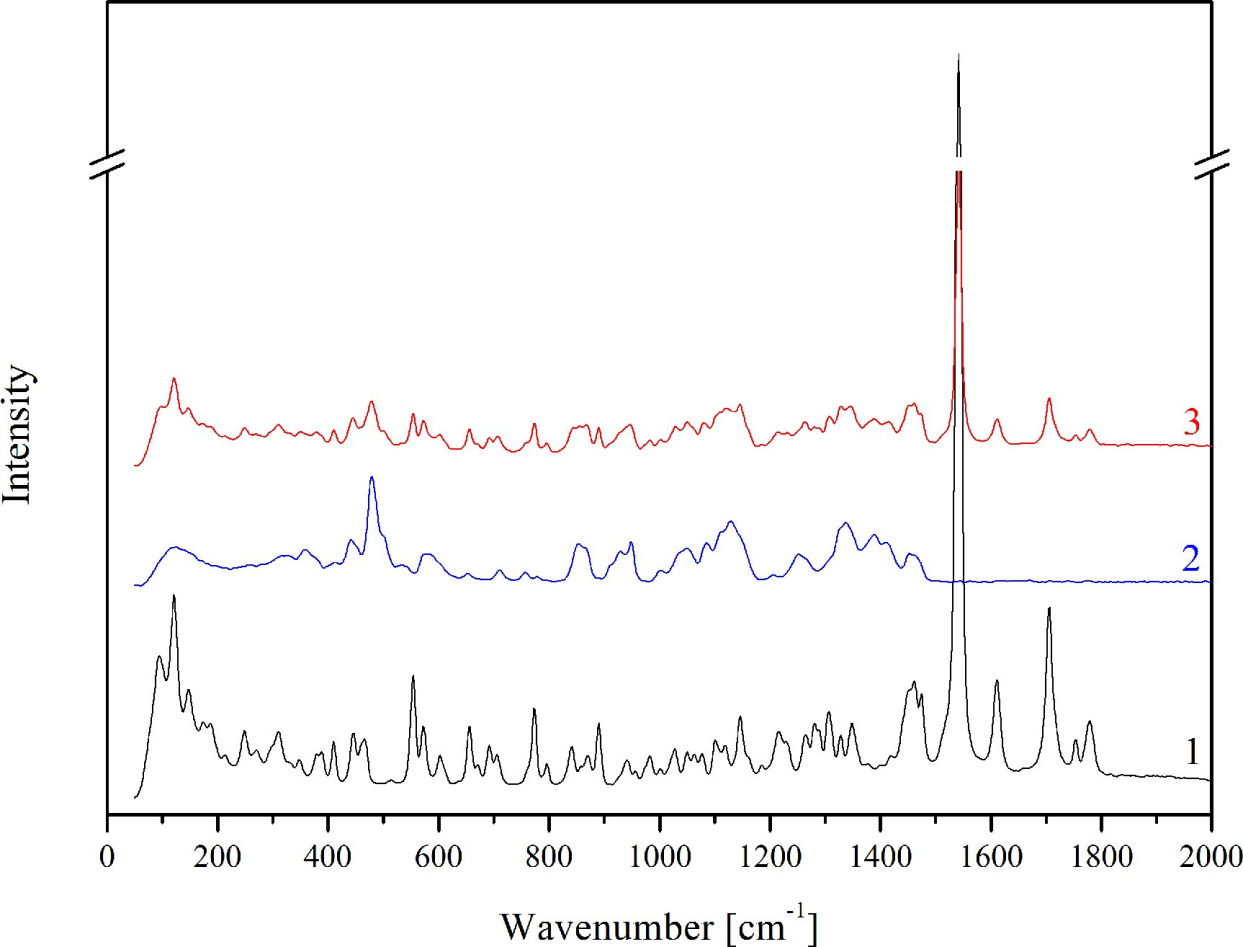
Raman spectra for TP (1), β-CD (2) and TP-β-CD inclusion complex (3).

Analysis of the types of bands, their location and their intensity of TP in a free form were obtained and compared with the theoretical spectra based on the quantum-chemical calculations performed with the use of B3LYP functional and 6-31G(d,p) basis set. The bands characteristic for TP, based on theoretical calculations, were described in our previous paper [28]. An analysis of changes in the TP-β-CD spectrum indicates mainly hydrogen interactions between the functional groups in the cyclodextrins and carbonyl and methyl groups in pivoxil substituent and possibly also hydrogen atoms of the bicyclic 4:5 rings, especially including the azetidine structure. The FT-IR absorption spectra in the region from 500 to 4000 cm^-1^ for TP (black line), β-CD (blue line) and the TP-β-CD complex (red line) were presented in Fig 1.The spectrum of TP-β-CD physical mixture was the sum of the TP and β-CD spectra and did not show any characteristic differences between bonds. The most characteristic differences of bands associated with hydrogen bonds which appeared in TP-β-CD were located at about 900 cm^-1^, between 1200 and 1800 cm^-1^ and at about 3000 cm^-1^. It was possible to identify the appearance of hydrogen bonds in these ranges, as an effect of a decrease in intensity of the following vibrations: C–O and CH_3_ stretching in the ester group at 855 cm^-1^, stretching between C–C, C–O and CH_3_ in the ester group at 1271 cm^-1^ and 1267 cm^-1^, C-H stretching and wagging and CH_3_ twisting in the ester group at 1449 cm^-1^, and C = O stretching in the carboxyl group and in the ester group at 1645 cm^-1^ and 1705 cm^-1^. Moreover, a decrease in intensity and a change in shape were observed for stretching vibrations of C-H in the thiazole ring and in the ester group at 2950 cm^-1^ and 2964 cm^-1^_,_ respectively. In Raman spectra, the significant varying intensities and shapes of the bands of TP-β-CD were observed at 1514 cm^-1^ and 2950 cm^-1^. They were mainly associated with the wagging, twisting and stretching vibrations of the CH_3_ and C-H bonds, respectively (Fig 2). The different intensities and shifts of the above-mentioned bands suggest the involvement of C = O and C–O bonds as well as the CH_3_ group in the pivoxil substituent in the formation of hydrogen bonds. Due to the significant susceptibility of the carbonyl group to hydrogen bonding, a similarly 4-fold reduction in the intensity of the bands of C-H bonds in the *trans*-hydroxyethyl substituent and the β-lactam ring (1075 cm^-1^) as well as the C-N band was noted for meropenem in complex with β-CD [27]. In the case of indomethacin after complexation, total disappearance of the carbonyl band was observed (1680 cm^-1^) [29].

### DSC analysis

**Fig 3:**
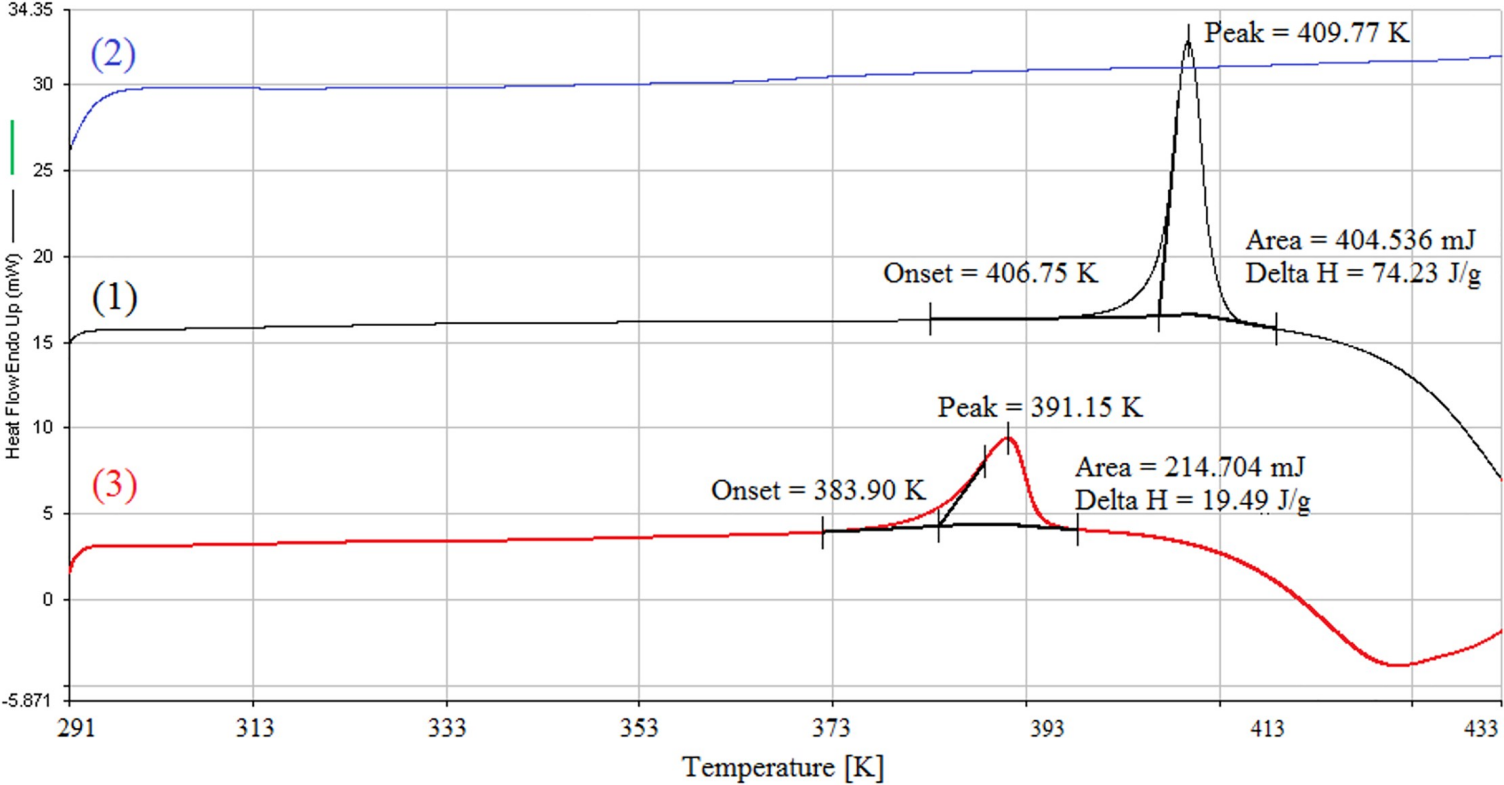
DSC thermograms of TP (1), β-CD (2) and TP-β-CD inclusion complex (3).

The DSC thermograms collected for TP, β-CD and the TP-β-CD inclusion complex are presented in Fig 3. DSC thermograms obtained for the pure TP demonstrated a sharp endothermic peak with a minimum at T = 409.77 K, which may be related to the melting point of the TP sample. Interestingly, no endothermic peak corresponding to the fusion of TP (at T = 409.77 K) was found in the DSC profile of TP-β-CD complex. In contrast, an endothermic peak with the marked broadening of less intensity (ΔH = 19.49 J g^-1^) was detected in the position of thermal transition of β-CD. The endothermic peak at T = 391.15 K is due to the shift of melting point of TP toward lower temperatures as a result of molecular encapsulation of TP into the β-CD nanocavity [30]. Moreover, the observed broadening of the TP-β-CD peak could also result from the phenomenon of masking of the TP melting endotherm and/or the fusion between TP melting and β-CD thermal transition due to the overlapping vicinity of the two effects; a similar phenomenon has been reported by Eida *et al*. [31].

### Theoretical calculations

**Fig 4:**
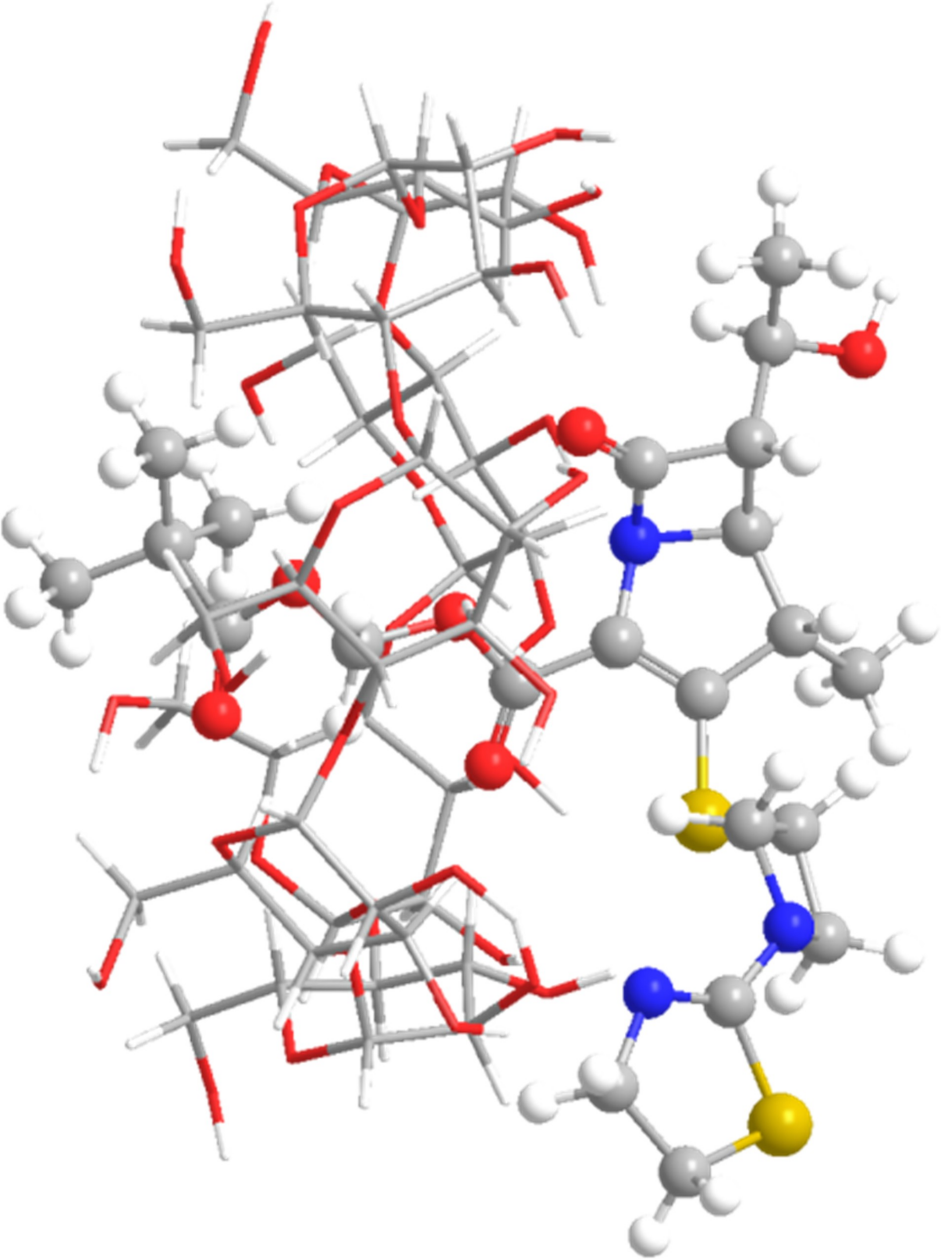
The conformation of the most stable complex of TP and β-CD.

*In silico* studies also confirmed that selected domains of pivoxil substituent are directly involved in interactions characteristic for “*guest–host*” complexes. Analysis of molecular dynamics simulations suggested the inclusion of pivoxil group into the β-CD cavity as the most probable mechanism of complex creation. The affinity of TP into β-CD was estimated to -22.0 kJ mol^-1^ (Fig 4). The most stable conformer is complex, where TP is not rotated relative to β-CD in respect to position acquired form molecular docking (Table 1). This conformer, according to the PM6 method, was also stable on the DFT/B3LYP 6-31G(d,p) level of theory. TP-β-CD interaction energy of conformation optimized with DFT/B3LYP corrected by basis set superposition error was -28.24 kJ mol^-1^.

**Table 1:** Relative energies of TP-β-CD complexes in respect to the angle of rotation of guest molecule rotated in the cavity.

Relative rotation [°]	0	30	60	90	120	150	180
Conformer/energy [A.U.]	0	+0.051	+0.029	+0.046	+0.029	+0.029	+0.029

Summarising, spectroscopic and thermal methods were used to confirm the identity of the complexes and their distinction from physical mixtures. It should be stressed that the use of the empiric methods and theoretical approach allowed to propose the domains of tebipenem (pivoxil substituent) involved in the interaction with the cyclodextrins.

**Fig 5:**
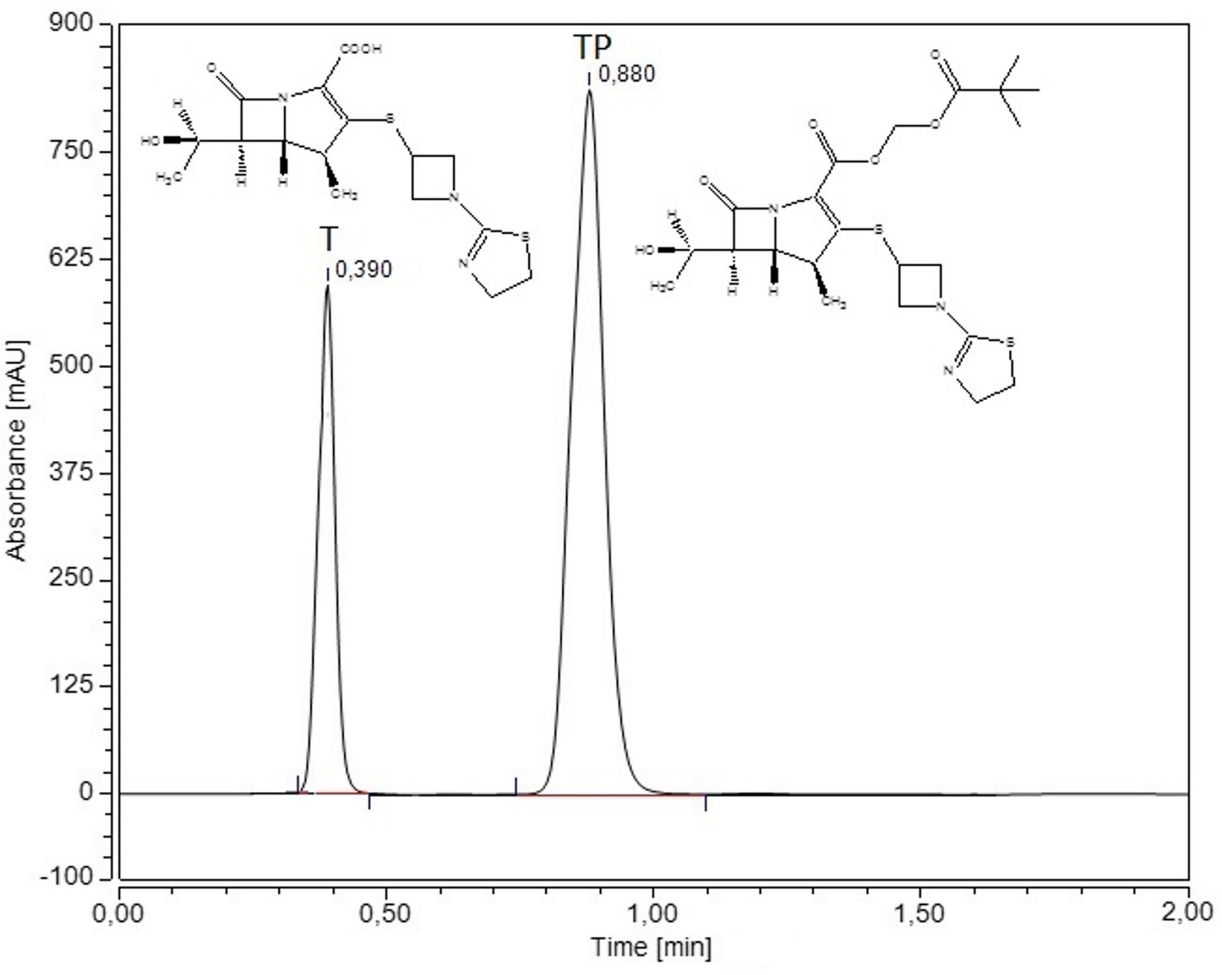
The chromatogram of TP in the presence its of the main impurity—tebipenem.

The second part of the study focused on the physicochemical (solubility, dissolution, chemical stability) and biological (permeability through artificial biological membranes and microbiological activity) properties after the inclusion of TP into the β-CD cavity. The physicochemical properties of TP-β-CD were evaluated with the use of the HPLC-DAD method developed for the determination of TP in the presence of its main impurity, tebipenem (Fig 5). The changes of tebipenem pivoxil concentrations in the presence of the main impurity, tebipenem, were evaluated using the HPLC-DAD method. The HPLC-DAD method was validated for this order in water, gastric juice (pH ~ 1.2) and in phosphate buffer (pH ~ 7.2), according to ICH guidelines (Table 2) [32]. According to data in the literature, TP is susceptible to degradation when exposed to physicochemical factors in aqueous solutions and in the solid state [13]. Depending on affecting factors, different degradation products were formed; therefore, the isocratic, short HPLC-DAD method was compared to the HPLC-DAD gradient method in order to confirm the selectivity of determination of the main analyte [33]. The biological property, such as permeability of the TP-β-CD complex through the Caco-2-cell monolayer, was determined by using the HPLC-DAD when changes in the TP concentrations in acceptor and donor solvents were monitored. During studies of the antibacterial activity of TP-β-CD, the minimal concentration of the system that inhibited strain growth was established, defined as the MIC, by the application of a well plate method.

**Table 2:** Validation parameters for HPLC-DAD method.

	Stability studies	Dissolution studies		Permeability studies	
Parameter	water	gastric juice (pH 1.2)	phosphate buffer (pH 7.2)	donor (pH 7.4)	acceptor (pH 7.4)
Selectivity					
Peak symmetry factor (0.8–1.5 required)	1.17	1.12	1.09	1.11	1.11
The absence of interfering substances	confirmed	confirmed	confirmed	confirmed	confirmed
Limit of detection LOD = 3 SD/a [mg mL^-1^]	0.0446	0.0261	0.0085	0.0153	0.0153
Limit of quantification LOQ = 10 SD/a [mg mL^-1^]	0.1351	0.0789	0.0256	0.0464	0.0464
Linearity y = ax + b					
a	17.05 ± 0.28	30.88 ± 0.25	1.18 ± 0.01	61.19 ± 4.01	61.19 ± 4.01
b	insignificant	insignificant	insignificant	insignificant	insignificant
Correlation coefficient (r)	0.9998	0.9999	0.9998	0.9965	0.9965
Range of linearity [mg mL^-1^]	0.01–1.00	0.04–2.40	0.04–1.44	0.02–1.20	0.02–1.20
Accuracy					
Recovery (95–105% required) [%]	95.56	101.25	102.27	95.00	95.00
Precision					
Concentration [mg mL^-1^]	0.45	2.40	0.44	0.20	0.20
Average of 6 injections [mg mL^-1^]	0.43	2.43	0.45	0.19	0.19
SD	0.01	0.04	0.01	0.002	0.002
RSD (<5% required) [%]	2.32	1.65	2.22	1.05	1.05

Where SD is the average of standard deviations of determinations in the lower range of linearity and a is the directional coefficient of the plotted linear function; S_*a*_ standard deviation of slope; S_*b*_ standard deviation of intercept, *t*, calculated values of Student’s t-test, t_α,f_ = 2.228 critical values of Student’s test for degrees of freedom f = 10 and significance level α = 0.05

### Phase-solubility studies

**Fig 6:**
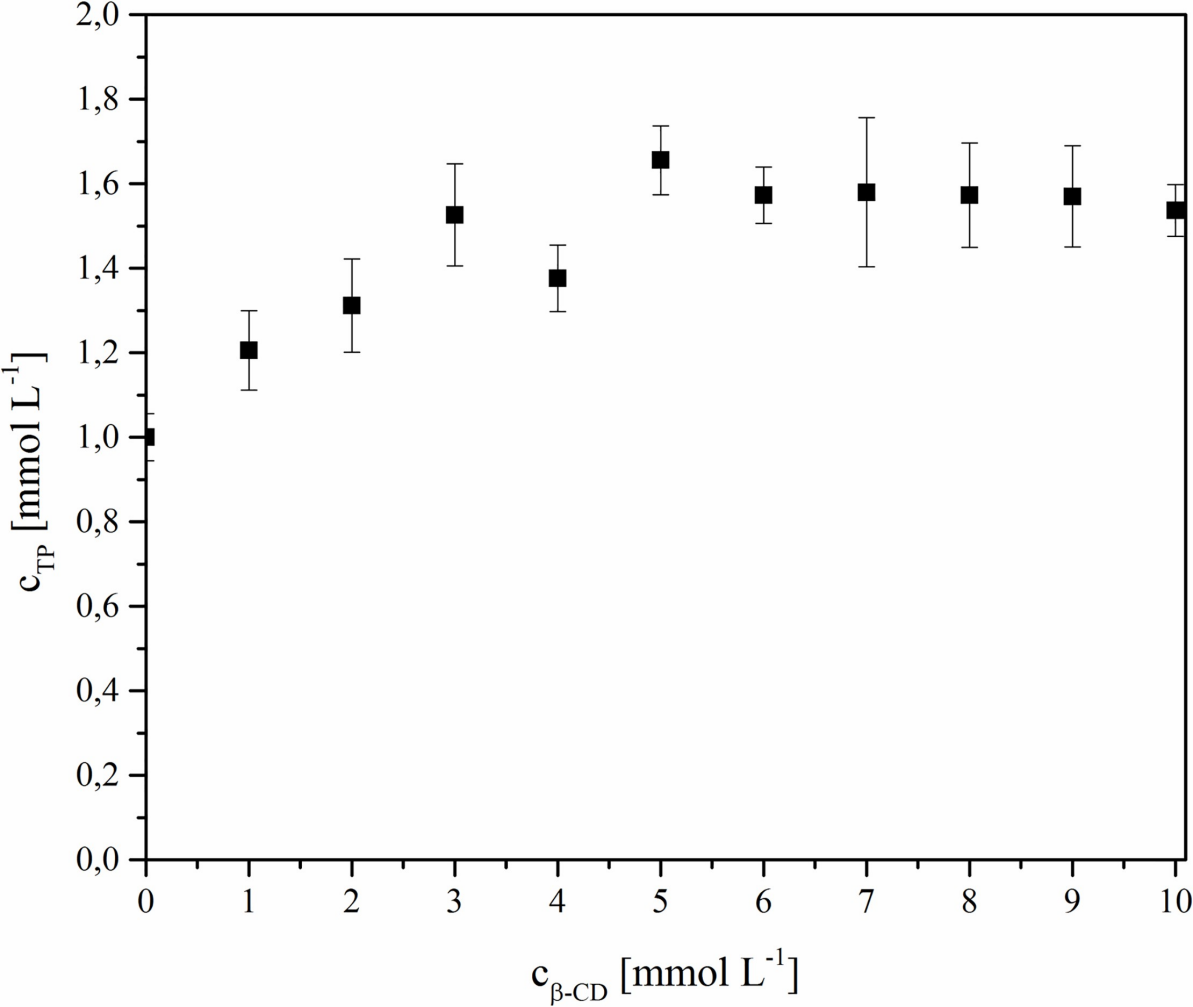
Phase-solubility diagram of TP inclusion complex in water.

An increase in TP solubility as a function of CD concentration was observed in the range of 0–3 mmol L^-1^. This is an effect of blocking a lipophilic pivoxil substituent as a result of the inclusion in β-CD and, at the same time, an increase in the number of hydroxyl groups connected with a β-CD solubility effect (Fig 6). Similar effects were observed for other APIs with a lipophilic character after connecting them with selected CDs [34]. A slightly different situation was observed in the case of another carbapenem analog, meropenem, on which the solubility influence of β-CD was not as significant, which can be explained by its stronger hydrophilic properties [18]. For estimation of the apparent stability constants, K_1:1_, parameters of the linear part of the solubility diagrams were used. The calculated stability constant was K_1:1_ = 0.1539 M^-1^ for the TP-β-CD complex (Fig 6).

### Dissolution studies

**Fig 7:**
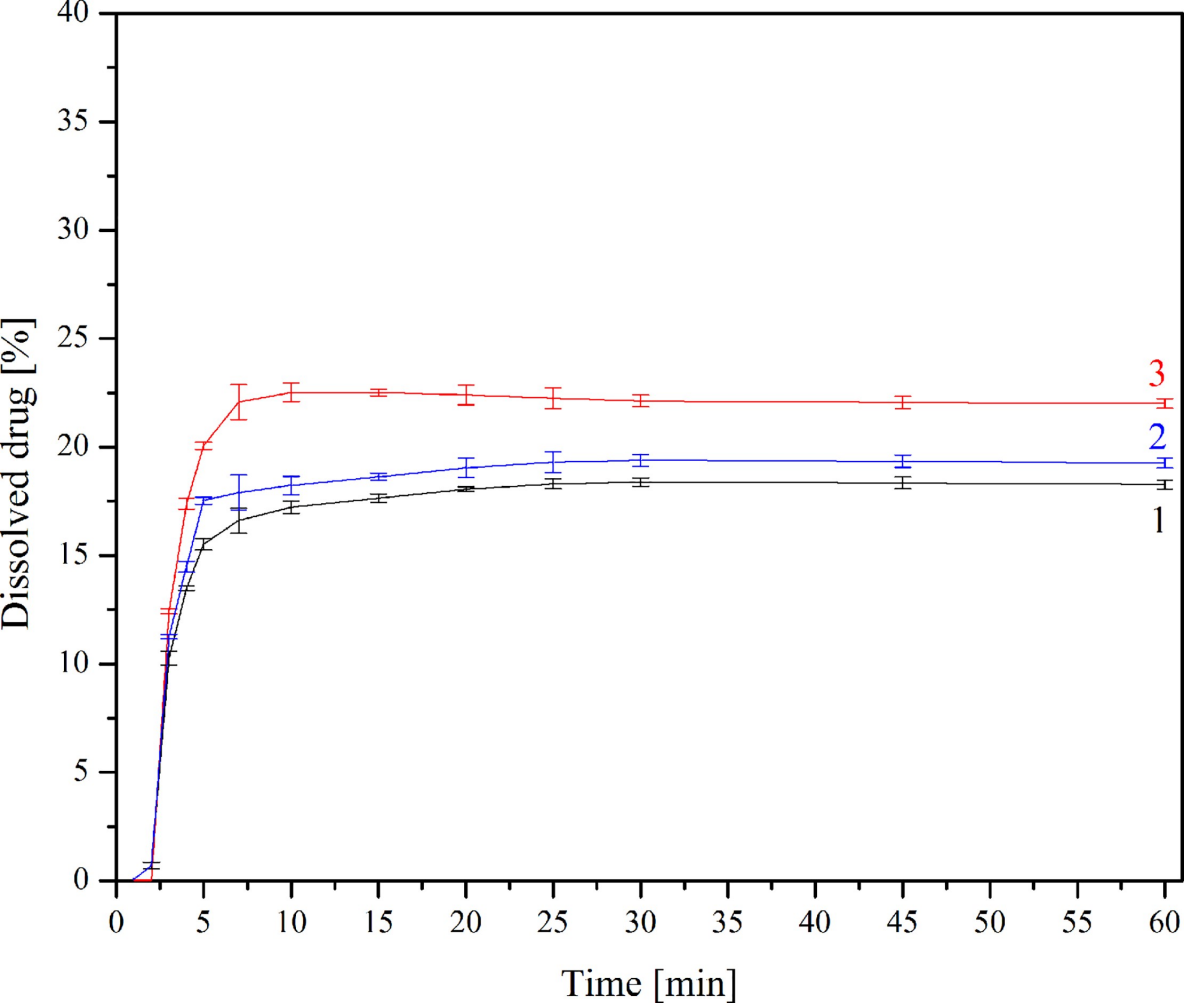
Dissolution profiles for TP (1), TP-β-CD physical mixture (2) and TP-β-CD inclusion complex (3) in artificial gastric juice at pH 1.2.

**Fig 8:**
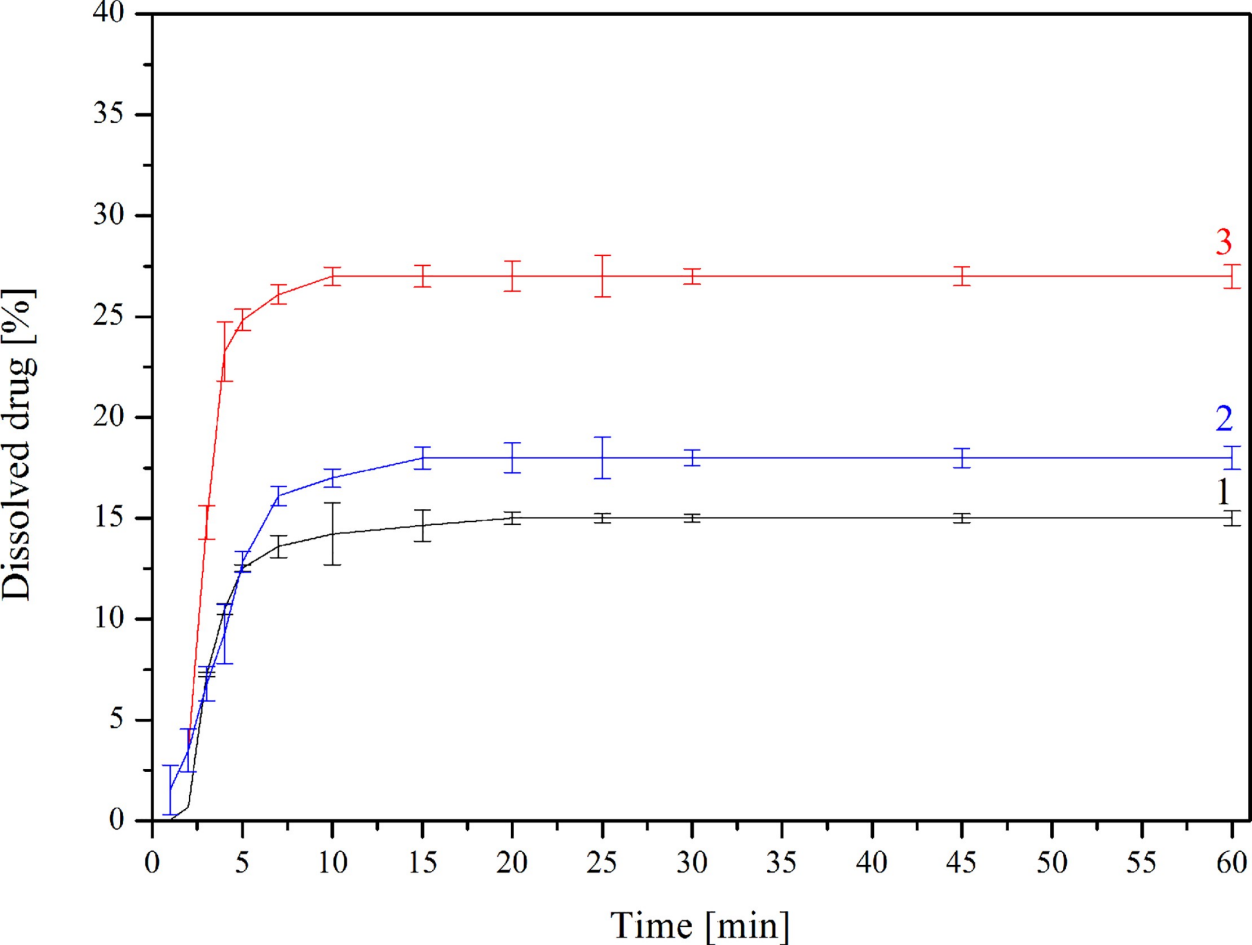
Dissolution profiles for TP (1), TP-β-CD physical mixture (2) and TP-β-CD inclusion complex (3) in phosphate buffer at pH 7.2.

The changes in dissolution rate of TP in a free, as well as complexed form, confirm a positive solubility effect of CDs; however, the presence of β-CD did not change the dissolution profile shape (Figs 7 and 8). Calculated *f*_1_ and *f*_2_ values confirmed that the dissolution profile of TP-β-CD complex is very different from pure TP in both dissolution media (*f*1/*f*2 values: 25.43/45.09 and 90.62/21.39 at pH 1.2 and 7.2, respectively). Interestingly, the differences in dissolution profiles were nearly twice as big (~ 12.5% vs. 7%) during research where phosphate buffer (pH ~ 7.2) was used as an acceptor fluid. Such different dissolution profiles show the potential of CDs as substances which significantly modify TP release, especially in the intestinal environment. Taking into consideration the prodrug character of TP, this observation becomes especially important as its proper absorption should take place in the intestines.

### Stability studies

The degradation of free TP, as well as TP after complexation with β-CD at an increased relative air humidity (303–333 K) and in dry air (333–363 K), was a first-order reaction depending on the substrate concentration; this is described by the following Eq 6:

$$\ln\left(C_{TP}\right)=\ln{\left(C_{TP}\right)}_{0}-k_{obs}$$ (6)

**Fig 9:**
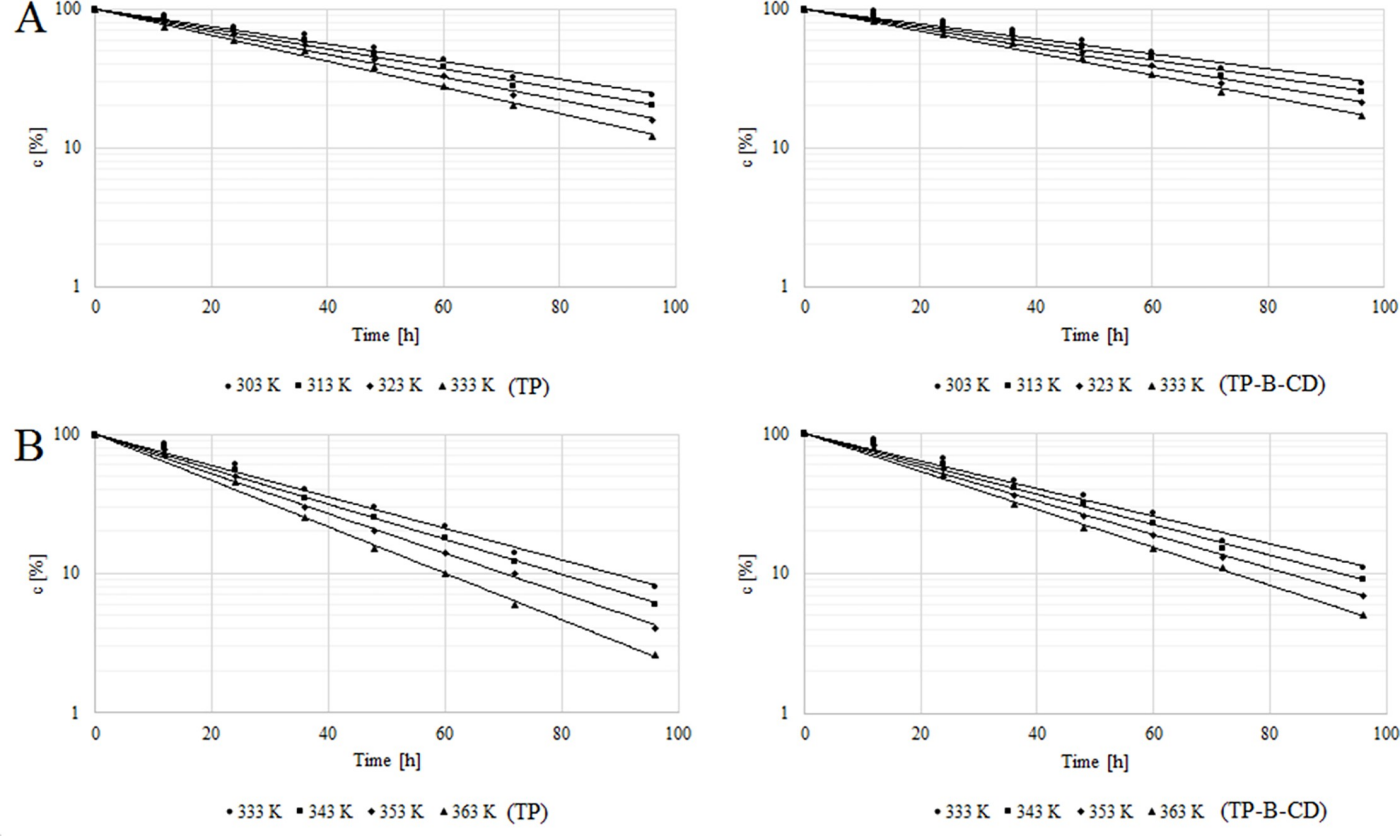
The semi-log plots of c = f(t) for degradation of TP and TP-β-CD inclusion complex studies at an increased relative air humidity (RH ~ 76%, T = 303–343 K) (A) and in dry air (RH = 0%, T = 333–363 K) (B).

The semi-logarithmic plots were linear and their slopes were equal to the rate constants of the reactions with the negative sign (-k_obs_). The test of the parallelism of slopes between the degradation plot of TP and TP-β-CD was used to resolve whether the k_obs_ determined for the degradation of TP and TP-β-CD was statistically significant (Table 3). Similarly to the rest of the carbapenems, humidity was a significant factor for tebipenem pivoxil degradation in the solid state [5]. However, a stronger stabilizing effect of β-CD in conditions of accelerated degradation in dry air in the case of the studied complex should be associated with a protective action towards pivoxil substituent as a result of its inclusion in β-CD. Previous research using tebipenem pivoxil showed many different degradation pathways depending on the conditions used in the research into accelerated degradation [13]. The lack of protective action towards a β-lactam bond in the studied TP-β-CD system results in smaller differences in the observed rate constants shown in the research into durability carried out at an increased relative air humidity, where the participation of hydrolysis as a degradation reaction is higher. The complexing of TP, however, did not significantly change the influence of temperature on the speed of its degradation. The relationship between the reaction rate constants and the temperature is described by Arrhenius Eq 7:

$$lnk_{i}=lnA-\frac{E_{a}}{RT}$$ (7)

where *k*_*i*_ is the reaction rate constants of TP [s^-1^], *A* denotes the frequency coefficient, *E*_*a*_ indicates activation energy [J mol^-1^], *R* is the universal gas constant [8.3144 J K^-1^ mol^-1^] and *T* represents temperature [K]. The straight-line relationship was obtained for TP and TP-β-CD at an increased relative air humidity (RH ~76%) in the temperature range 303–333 K and in dry air in the range 333–363 K. The least squares method was used to calculate the slopes (a) and frequency coefficient (ln*A*), which allowed the calculation of activation energy, enthalpy (Δ*H*^≠a^) and entropy (Δ*S*^≠a^) at 298 K (Table 3, Fig 9). For both studied degradation conditions, similar values of activation energy, as well as enthalpy and entropy, were observed. Negative values of ΔS^≠^ indicate a dimolecular degradation of tebipenem pivoxil in both degradation conditions. The presence of an ester bond into the structure of carbapenem analogs resulted in cyclodextrin inclusion of that ester bond. In our previous work, in the case of the acidic carbapenem analog meropenem, we observed above all the inclusion of trans-hydroxyethyl substituent into β-CD [18]. The previously obtained results explained a stabilizing influence of β-CD on the durability of meropenem in the solid state at an increased relative air humidity where hydrolysis of the β-lactam bond is the principal degradation process [18]. Therefore, we should now indicate the inclusion of pivoxil substituent into β-CD as the most essential cause of TP stabilizationin the solid state under thermolysis conditions.

**Table 3:** Kinetic and thermodynamic parameters of the degradation of TP in free form and in complex with β-CD.

Temp. [K]	TP		TP-β-CD inclusion complex		t_o_
	(k ±Δk) [s^-1^]	Thermodynamic parameters (mean ± SD)	(k ±Δk) [s^-1^]	Thermodynamic parameters (mean ± SD)	
RH ~ 76%					
303 313 323 333	(3.94 ± 0.52) × 10^−6^ (4.80 ± 0.40) × 10^−6^ (5.38 ± 0.38) × 10^−6^ (6.12 ± 0.42) × 10^−6^	*E*_*a*_ = 12.07 ± 3.80 (kJ mol^-1^) *ΔH*^*≠*a^ *=* 9.59 ± 6.28 (kJ mol^-^) *ΔS*^*≠*a^ *=* -244.91 ± 209.78 (J K^-1^ mol^-1^)	(3.69 ± 0.52) × 10^−6^ (4.22 ± 0.45) × 10^−6^ (4.67 ± 0.39) × 10^−6^ (5.22 ± 0.36) × 10^−6^	*E*_*a*_ = 9.59 ± 1.18 (kJ mol^-^) *ΔH*^*≠*a^ *=* 7.11 ± 3.66 (kJ mol^-^) *ΔS*^*≠*a^ *=* -244.92 ± 231.91 (J K^-1^ mol^-1^)	1.72 2.34 3.14 3.96
RH = 0%					
333 343 353 363	(7.64 ± 0.02) × 10^−6^ (8.39 ± 0.01) × 10^−6^ (9.42 ± 0.02) × 10^−6^ (1.09 ± 0.02) × 10^−5^	*E*_*a*_ = 11.84 ± 4.51 (kJ mol^-^) *ΔH*^*≠*a^ *=* 9.36 ± 6.98 (kJ mol^-^) *ΔS*^*≠*a^ *=* -244.91 ± 205.34 (J K^-1^ mol^-1^)	(6.80 ± 0.03) × 10^−6^ (7.32 ± 0.02) × 10^−6^ (7.98 ± 0.02) × 10^−6^ (8.85 ± 0.02) × 10^−6^	*E*_*a*_ = 8.79 ± 2.73 (kJ mol^-^) *ΔH*^*≠*a^ *=* 6.31 ± 5.21 (kJ mol^-^) *ΔS*^*≠*a^ *=* -244.92 ± 223.55 (J K^-1^ mol^-1^)	2.35 3.67 5.02 6.62

*Δk = S*_*a*_ *t*_*α*_*f E*_*a*_, activation energy; *ΔH*^*≠*^, enthalpy; *ΔS*^*≠*^, entropy; *Ea = -aR*; *ΔH*^*≠*^ *= E*_*a*_ *-TR*; *ΔS*^*≠*^ *= R(ln A ln(k*_*b*_*T)/h* where: k_B_, Boltzmann’s constant (1.3807 10^−23^ JK^-1^); *h*, Planck’s constant (6.626 10^-34^Js); *R*, universal gas constant (8.314 K^-1^mol^-1^), *T*, temperature in K; a, vectorial coefficient of the Arrhenius; *A*, frequency coefficient *a* calculated for 298 K; t_0_, parameter of parallelism test, establishing significance of *a* coefficient of ln(c_i_) = f(t) plots.

Based on results showing the changes in the physicochemical properties of TP after its inclusion into the cavity of β-CD (an increased solubility, dissolution and chemical stability), biological implications such as permeability through artificial biological membranes and microbiological activity were studied.

### Permeability studies

The Caco-2 cell permeability test was considered a reliable tool for screening the transport efficiency of new drugs and formulations through artificial biological membranes [34]. Therefore, in our study, the permeability of TP across Caco-2 cells was investigated for pure TP and the TP-β-CD inclusion complex. The permeability coefficient values (P_app_ (A→B) and P_app_ (B→A)) and efflux ratio of pure TP and in CD complex are shown in Table 4. Generally, substances with an apparent permeability coefficient (P_app_) of less than 1×10^−6^ cm s^-1^ are classified as low permeability substances. Medium permeability substances have P_app_ values between 1×10^−6^ and 1×10^−5^ cm s^-1^, and high permeability substances exhibit apparent permeability coefficients of >1×10^−5^ cm s^-1^ [35]. To verify that P_app_ determined for permeability of tebipenem pivoxil in free and complexed forms was statistically different, ANOVA test was used.

**Table 4:** The permeability values and efflux ratios of pure TP and TP-β-CD inclusion complex.

	P_app_ (A→B)	P_app_ (B→A)	Efflux ratio P_app_ (B→A) / P_app_ (A→B)
	(mean ± SD) x 10^−6^ [cm s^-1^]		
pure TP	40.22 ± 0.51	75.14 ± 0.64	1.87
TP-β-CD complex	5.28 ± 0.01	8.96 ± 0.02	1.70

where P_app_ (A→B)—permeability value for A → B direction, P_app_ (B→A)—permeability value for B → A direction; A—donor, B—acceptor

CDs have been suggested to perform as drug carriers to the gastrointestinal membrane and to enhance the penetration of drugs into the intestine [36]. According to the obtained results, the permeability value (P_app_ (A→B)) for pure TP was found to be 40.22×10^−6^ cm s^-1^; however, the permeability value (P_app_(A→B)) for the TP-β-CD complex was much lower (statistical difference, p < 0.05). It can be concluded that TP-β-CD did not promote permeability through the Caco-2 cell monolayer. Despite that, the effects of β-CD on the TP efflux across Caco-2 cell monolayers were investigated. The efflux ratio of pure TP was about 1.87 while of TP from the TP-β-CD complex decreased to 1.70. Kato *et al*. confirmed the participation of given protein carriers (OATP1A2 and OATP2B1) in the absorption of tebipenem pivoxil [12]. The implementation of lipophilic substituents in β-lactam antibiotics from penam, cephem, and carbapenem groups is commonly associated with the growth of their bioavailability [1]. The decrease in the permeability of TP after its inclusion into the cavity of β-CD may be an effect of blocking the pivoxil substituent delivering tebipenem responsible for the increased lipophilic character. However, on the basis of the obtained results, at the same time, it can be suggested that there are changes to the pivoxil substituent domain interactions with protein carriers as a result of the emergence of the TP-β-CD complex. Nevertheless, the hydrophilic nature of the TP-β-CD complex cannot be associated with the increase in passive transport, mostly characterizing drug permeation (including drug delivery) [37]. Summarising this stage of the work, the authors will refer to the decrease in intensity of the efflux phenomenon while discussing the changes in the antibacterial activity of the TP-β-CD complex.

### Antibacterial activity studies

While estimating the antibacterial activity of the TP-β-CD complex, a minimal inhibitory concentration was defined for the growth of 10 bacteria species, including Gram-positive and Gram-negative species. The antibacterial activity of tebipenem in a free form was defined as a reference standard. As literature suggests, tebipenem pivoxil shows activity against methicillin-resistant *Staphylococcus aureus* (MRSA), methicillin-resistant *Staphylococcus epidermidis*, *Enterococcus faecalis*, *Escherichia coli*, *Klebsiella pneumoniae*, *Enterobacter aerogenes* and *Pseudomonas aeruginosa* [8]. The inclusion of tebipenem pivoxil domains did not reduce its antibacterial activity in relation to any of the studied bacteria strains, which can be explained by the lack of detrimental changes in the β-lactam bond which is equated to the antibacterial activity of β-lactam antibiotics (Table 5). Moreover, in the case of the TP-β-CD complex, MIC values were significantly reduced in relation to growth inhibition of *Staphylococcus aureus*, *Enterococcus faecalis*, *Pseudomonas aeruginosa* and *Proteus mirabilis*. Taking into consideration that all of the above-mentioned microorganisms cause serious hospital-acquired and community-acquired infections that are frequently difficult to cure due to the emergence of resistant strains, the authors searched for an explanation for significant decrease in MIC values characterizing the antibacterial potential of the TP-β-CD complex. The decrease in MICs needed to inhibit the growth of *Staphylococcus aureus* was quadruple in the case of the TP-β-CD complex (the MIC value was 15 mgL^-1^ for free TP and 4 mg L^-1^ for the TP-β-CD complex), but was double for *Enterococcus faecalis*(250 mg L^-1^vs 125 mg L^-1^), *Pseudomonas aeruginosa* (250 mg L^-1^ vs 125 mg L^-1^) and *Proteus mirabilis* (125 mg L^-1^ vs 62 mg L^-1^ –clinical isolate; 31 mg L^-1^ vs 15 mg L^-1^ –reference strain). Increasing antibacterial resistance and the appearance of new isolated pathogens requires a novel approach to treat *S*. *aureus*-mediated infection, including methicillin-resistant *S*. *aureus* (MRSA). One of the significant factors defined as a virulence factor consists of the production of α-hemolysin (also known as α-toxin) by *S*. *aureus*. Based on a crystallographic analysis carried out in earlier research, it was observed that there were similarities between the diameter of the α-hemolysin channel lumen (14–46 Å) and the external diameter of β-CD (15.3 Å) [38]. In the literature, information can be found about several compounds (including β-CD) which inhibit the α-hemolysin cytotoxicity of *S*. *aureus*. A few causes were found when β-CD derived protected the cell against α-hemolysin [39]. In our case, a similar situation may take place; a created TP-β-CD complex acts with its cyclodextrin part and blocks porins in *S*. *aureus*, preventing α-hemolysin from being released. At the same time not blocking the β-lactam bond in tebipenem contributes to the more efficient antibacterial activity of the TP-β-CD complex compared to a free antibiotic. An increase in antibacterial activity was also observed in the cases of cefdinir-β-CD and meropenem-β-CD complexes [40]. In the case of carbapenems, very important mechanisms of resistance are the production of plasmid- or integron-mediated carbapenemases, the increased expression of efflux effect, reduced porin expression and the overproduction of penicillin-binding proteins (PBPs) [17]. Taking into account a double decrease of MICs characterising the antibacterial activity of the TP-β-CD complex in relation to *Enterococcus faecalis*, *Pseudomonas aeruginosa* and *Proteus mirabilis*, it seems possible to suggest two mechanisms inhibiting a development of selected strains of the above-mentioned bacteria in the presence of the TP-β-CD complex. Firstly, blocking porin channels contributed to the *efflux* effect in bacteria by CDs. Limitations of efflux transport in the case of the TP-β-CD complex was observed by the authors as early as the stage of the permeability studies. The efflux ratio of pure TP was about 1.87, while that of TP from the TP-β-CD complex decreased to 1.70 when the model of Caco–2 cell monolayers was used. Due to the previously confirmed fact that zinc atoms and native cyclodextrins including β-CD create complexes, the other mechanism might be interactions between β-CD and zinc atoms forming the active site in carbapenemases [41–44]. “Blocking” the zinc atoms prevents them from participating in the hydrolysis of bicyclic β-lactam rings, which may also contribute to the antibacterial activity of the TP-β-CD complex.

**Table 5:** Values of MIC (mg L^-1^) of TP and TP-β-CD inclusion complex against selected Gram-positive and Gram-negative bacteria.

Microorganism		MIC (mg L^-1^)	
		pure TB	TP-β-CD
1	Salmonella typhimurium*	125	125
2	*Salmonella typhimurium* ATCC 14028	125	125
3	*Listeria monocytogenes*	125	125
4	*Listeria monocytogenes* ATCC 7644	125	125
5	Staphylococcus aureus*	31	15⇩
6	*Staphylococcus aureus* ATCC 25923	15	4⇩
7	Klebsiella pneumoniae*	125	125
8	*Klebsiella pneumoniae* ATCC 31488	31	31
9	Pseudomonas aeruginosa*	250	125⇩
10	*Pseudomon saeruginosa* ATCC 27853	250	125⇩
11	Proteus mirabilis*	125	62⇩
12	*Proteus mirabilit* ATCC 12453	31	15⇩
13	Enterobacter aerogenes*	250	250
14	*Enterobacter aerogenes* ATCC 13048	62	62
15	Enterobacter hormaechei*	125	125
16	*Enterobacter hormaechei* ATCC 700323	125	125
17	Enterococcus faecalis*	250	125⇩
18	*Enterococcus faecalis* ATTC 29212	250	125⇩
19	Alcaligenes faecalis*	125	125
20	*Alcaligenes faecalis* ATCC 29212	125	125

*clinical isolates

⇩signs lower MIC values for TP-β-CD

## Conclusion

The creation of the first oral carbapenem analog complex with β-CD constitutes an example of the possible modification of physicochemical and biological properties that are important from the perspective of designing modern oral forms as well as searching for a new approach for obtaining effective antibiotic systems in the era of widespread antibiotic resistance. On the basis of the received results, as the most significant achievements in the field of modifying the physicochemical properties of tebipenem pivoxil, the authors consider: (i) identifying tebipenem pivoxil domains directly engaged in inclusion into β-CD, (ii) proving an essential modification of the tebipenem pivoxil dissolution rate as a result of interactions with β-CD, especially in the environment simulating intestinal matter, which is a location of prodrug absorption, and (iii) proving a stabilising effect of β-CD in relation to tebipenem pivoxil in the solid state, especially under conditions of thermolysis. The most significant biological effects of tebipenem pivoxil after its complexation by β-CD are the decreased absorption of tebipenem pivoxil by the model of Caco-2 cell monolayers and increased antibacterial activity in relation to bacterial strains (*Staphylococcus aureus*, *Pseudomonas aeruginosa*, *Enterococcus faecalis*, *Proteus mirabilis*) which are considered clinically difficult to cure. In summary, the presented approach constitutes a solution with effective and viable antibacterial potential, as well as physicochemical properties required for innovative drug delivery. The presented approach to curing resistant clinical infections in the current situation of searching for new chemotherapeutics also has an essential advantage of the safe and secure application of the involved substances.
